# Post-operative paediatric cerebellar mutism syndrome: time to move beyond structural MRI

**DOI:** 10.1007/s00381-018-3867-x

**Published:** 2018-06-20

**Authors:** Sebastian M. Toescu, Samantha Hettige, Kim Phipps, RJ Paul Smith, Verity Haffenden, Chris Clark, Richard Hayward, Kshitij Mankad, Kristian Aquilina

**Affiliations:** 1 Department of Neurosurgery, Great Ormond Street Hospital, London, WC1N 3JH UK; 2 Developmental Imaging and Biophysics Section, Institute of Child Health, 30 Guilford Street, London, WC1N 1EH UK; 3 Department of Neuroradiology, Great Ormond Street Hospital, London, WC1N 3JH UK

**Keywords:** Posterior fossa syndrome, Cerebellar mutism syndrome, Structural MRI, Medulloblastoma

## Abstract

**Purpose:**

To determine the value of structural magnetic resonance imaging (MRI) in predicting post-operative paediatric cerebellar mutism syndrome (pCMS) in children undergoing surgical treatment for medulloblastoma.

**Methods:**

Retrospective cohort study design. Electronic/paper case note review of all children with medulloblastoma presenting to Great Ormond Street Hospital between 2003 and 2013. The diagnosis of pCMS was established through a scoring system incorporating mutism, ataxia, behavioural disturbance and cranial nerve deficits. MRI scans performed at three time points were assessed by neuroradiologists blinded to the diagnosis of pCMS.

**Results:**

Of 56 children included, 12 (21.4%) developed pCMS as judged by a core symptom of mutism. pCMS was more common in those aged 5 or younger. There was no statistically significant difference in pre-operative distortion or signal change of the dentate or red nuclei or superior cerebellar peduncles (SCPs) between those who did and did not develop pCMS. In both early (median 5 days) and late (median 31 months) post-operative scans, T2-weighted signal change in SCPs was more common in the pCMS group (*p* = 0.040 and 0.046 respectively). Late scans also showed statistically significant signal change in the dentate nuclei (*p* = 0.024).

**Conclusions:**

The development of pCMS could not be linked to any observable changes on pre-operative structural MRI scans. Post-operative T2-weighted signal change in the SCPs and dentate nuclei underlines the role of cerebellar efferent injury in pCMS. Further research using advanced quantitative MRI sequences is warranted given the inability of conventional pre-surgical MRI to predict pCMS.

## Introduction

Post-operative paediatric cerebellar mutism syndrome (pCMS) is a well-recognised complication of resective surgery for brain tumours of the cerebellum and fourth ventricle region in children. Originally described as ‘akinetic mutism’ [1] in the setting of posterior fossa surgery in 1958, and more widely recognised by the mid 1980s [2–4], ‘cerebellar mutism syndrome’ or ‘posterior fossa syndrome’ has been described following infectious [5, 6], traumatic [7, 8] or vascular [9, 10] brain pathologies. However, the majority of cases arise following craniotomy for infratentorial brain tumours in children, with an incidence in this group of patients reported between 24 and 39% [11, 12]. pCMS is characterised by a delayed onset of mutism and emotional lability around 24 h following surgery. It can also be associated with hypotonia and other cerebellar motor signs, cerebellar cognitive affective syndrome [13], long tract motor signs and cranial neuropathies [14]. Recovery usually occurs over 6 months, although most children are left with residual speech, motor and neurocognitive deficits [15, 16].

**Fig. 1:**
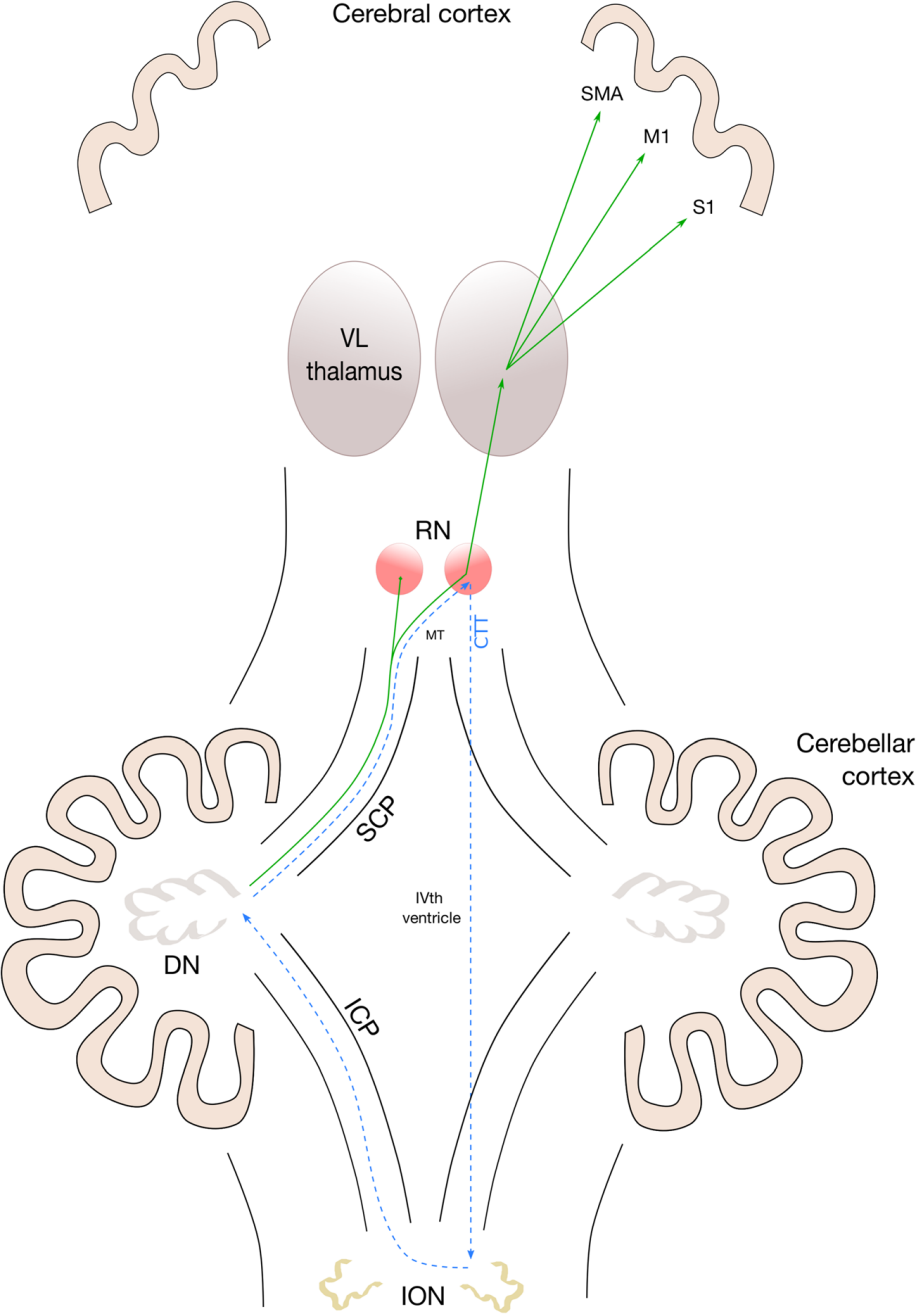
Schematic anatomical figure showing relationship of dentato-rubro-thalamo-cortical tract (DRTC, solid line) and triangle of Guillain-Mollaret (dotted line) in relation to cerebellar afferent and efferent pathways. CTT central tegmental tract, DN dentate nucleus, ICP inferior cerebellar peduncle, ION inferior olivary nucleus, M1 primary motor cortex, MT mesencephalic tegmentum, RN red nucleus, S1 primary sensory cortex, SMA supplementary motor area, VL thalamus ventrolateral nucleus of thalamus

There is a growing body of evidence, taken increasingly from advanced MRI studies such as diffusion tractography [17–20], which implicates the proximal efferent cerebellar pathway (pECP) as the anatomical substrate of pCMS [21]. The pECP comprises the dentate nucleus, superior cerebellar peduncle (SCP) and its decussation in the mesencephalic tegmentum as its fibres travel towards the red nucleus and on towards the thalamus. This functional bundle contributes intimately to the ‘triangle of Guillain-Mollaret’ (see Fig. 1), a brainstem feedback loop with hypothesised associations to pCMS. Yet beyond these largely structural associations, there is as yet no unifying aetiological hypothesis for the pathophysiology of this condition [21, 22], with its distinctive temporal onset and resolution.

A number of neuroimaging markers which can be seen on conventional pre-operative MRI have been associated with the development of pCMS. These include brainstem infiltration or compression [23–25], tumour histology and size [26], midline [27] and more rostral locations within the fourth ventricle [17]. However, none of these are currently able to accurately predict the occurrence of pCMS in a given patient. Our aim in this study was to re-evaluate the pECP to determine if any markers on pre-operative conventional MRI could predict development of pCMS and to study post-operative conventional MRI surrogates of efferent cerebellar damage in a cohort of children with medulloblastoma.

## Methods

We retrospectively interrogated a prospectively maintained neuro-oncology database and case note files to yield data on demographics, surgical technique and histopathology. Eligible participants were all children aged under 16 diagnosed with histologically confirmed medulloblastoma following surgical resection at our institution between 2003 and 2013 inclusive. The diagnosis of pCMS was established by means of a scoring system with core symptoms of mutism; behavioural disturbance and ataxia and additional features of dysphagia, dysmetria and other lower cranial nerve dysfunction. Histopathological samples were analysed by a team of specialist paediatric neuropathologists. Patients were routinely followed up in neuro-oncology multidisciplinary team meetings and clinics. Our institution does not require additional patient consent or ethical approval for such retrospective evaluations.

MR imaging was performed on a Siemens 1.5 Tesla or 3 Tesla scanner. T1-weighted, T2-weighted, fluid-attenuated inversion recovery (FLAIR) and diffusion-weighted (DWI) sequences were taken pre-operatively and within the first post-operative week. Delayed imaging, acquired for routine clinical follow-up, was also analysed. Analysis of the MR images was performed jointly by two senior neuroradiologists blinded to diagnosis of pCMS. Consensus was reached at the time of primary review. Diffusion-weighted sequences (*b* = 1000) were reviewed alongside derived apparent diffusion coefficient (ADC) maps for corroboration of positive findings.

Descriptive statistical analysis was performed and pre-surgical and clinical variables were analysed between patients with and without pCMS using λ-squared or student *t* test. Where relevant, Fisher’s exact test was used to compare radiological variables to ascertain statistically significant differences between those with and without pCMS. Statistical analysis was performed with IBM SPSS Statistics (IBM, Armonk, New York, Version 24, 2016). A prospectively determined *p* value of 0.05 was used to indicate a significant difference.

## Results

Fifty-six consecutive patients were included (34 male), with an age range from 2 months to 14 years (median 6 years 1 month). Twelve patients (21.4%) were found to have developed pCMS (see Table 1). There were no statistically significant differences in gender, age or size of tumour between the two groups. Subgroup analysis indicated a higher incidence of pCMS in patients aged 5 or younger, although the difference did not reach statistical significance (*p* = 0.099).

**Table 1:** Patient characteristic and clinical variables

	pCMS	No pCMS	*p*
No. of patients	12	44	
Gender male, *n* (%)	6 (50)	28 (64)	0.30*
Age at diagnosis, median (range)	4 years (7 months–10 years)	6 years (2 months–14 years)	0.22**
Transverse dimension of tumour (mm), median (range)	43.9 (21–69)	43.8 (15–84)	0.98**

*p values calculated using Fisher’s exact test

**p values calculated using Student *t* test

### Pre-operative imaging

There was no statistically significant difference between the two groups with regard to tumour size (*p* = 0.98). Although pre-operative changes of the dentate, red nuclei and SCP were seen in some patients who went on to develop pCMS, these differences between groups did not reach statistical significance (Table 2).

**Table 2:** Pre-operative MRI results

	pCMS	No pCMS	*p*
T2W red nuclei signal change			0.52
Bilateral None	1 11	2 42	
T2W dentate nuclei signal change			0.63
Bilateral None Unilateral	5 4 3	24 9 11	
T2W dentate nuclei distortion			0.62
Bilateral None Unilateral	10 2 0	34 5 5	
T2W SCP signal change			0.91
Bilateral None Unilateral	4 5 3	14 22 8	
T2W SCP distortion			0.75
Bilateral None Unilateral	10 2 0	30 9 5	
DWI/ADC dentate signal change			0.83
Bilateral None Unilateral	2 10 0	5 36 3	
DWI/ADC SCP signal change			0.44
Bilateral None Unilateral	0 12 0	4 35 5	

*p* values calculated using Fisher’s exact test

*ADC* apparent diffusion coefficient, *DWI* diffusion-weighted imaging; *SCP* superior cerebellar peduncle, *T2W* T2 weighted

### Post-operative imaging review

Post-operative scans were acquired in the first week after surgery (median 5 days). The dentate nuclei, SCP and red nuclei were again reviewed. There was a statistically significant difference in T2-weighted SCP signal change between patients who developed pCMS and those who did not (*p* = 0.040). No other differences on T2-weighted or DWI sequences were observed between groups (see Table 3).

**Table 3:** Post-operative MRI results

	pCMS	No pCMS	*p*
T2W red nuclei signal change			1.0
Bilateral None	0 12	2 42	
T2W dentate nuclei signal change			0.59
Bilateral None Unilateral	11 0 1	32 3 9	
T2W SCP signal change			0.040*
Bilateral None Unilateral	6 1 5	9 19 16	
DWI/ADC dentate nuclei signal change			0.68
Bilateral None Unilateral	5 6 1	14 22 8	
DWI/ADC SCP signal change			1.0
Bilateral None Unilateral	2 8 2	6 29 9	

*p* values calculated using Fisher’s exact test

*ADC* apparent diffusion coefficient, *DWI* diffusion-weighted imaging, *SCP* superior cerebellar peduncle, *T2W* T2 weighted

*Statistical significance at *p* = 0.05

On delayed follow-up imaging (median 31 months), there were significant differences in T2-weighted signal change in both dentate nuclei (*p* = 0.024) and SCP (*p* = 0.046) in patients who developed pCMS compared to those who did not (Table 4).

**Table 4:** Follow-up MRI results. T2-weighted sequence analysis

	pCMS	No pCMS	*p*
Red nuclei signal change			1.0
Bilateral None	0 12	1 43	
Dentate nuclei signal change			0.024*
Bilateral None Unilateral	10 0 2	20 15 9	
SCP signal change			0.046*
Bilateral None Unilateral	4 3 5	5 27 12	
ION signal change			0.85
Bilateral None Unilateral	2 9 1	8 34 2	

*p* values calculated using Fisher’s exact test

*ION* inferior olivary nucleus, *SCP* superior cerebellar peduncle

*Statistical significance at *p* = 0.05

## Discussion

pCMS is an increasingly well-recognised complication of infratentorial brain tumour resection in children. In this contemporary single-institution consecutive case series, we demonstrate an incidence of 21.4% in children undergoing craniotomy for resection of medulloblastoma. We were unable to demonstrate any significant difference in pre-operative structural MRI characteristics of the dentate nuclei, SCP or red nuclei between patients who did and did not develop pCMS. Review of post-operative structural MRI revealed statistically significant T2-weighted signal change in SCPs on scans within the first post-operative week, as well as statistically significant T2-weighted signal change in dentate nuclei and in SCPs on delayed follow-up scans in patients diagnosed with pCMS.

Symptoms consistent with what we now term pCMS were first reported by Daly and Love in 1958 following occipital craniectomy and trans-vermian resection of an astrocytoma in a 14-year-old boy. Many cite that of Rekate as the first description of the syndrome, in a six-patient case series in 1985 [4]. Since then, the nomenclature of this condition has gone through a series of developments, from ‘transient cerebellar mutism’, ‘mutism and subsequent dysarthria’, through posterior fossa syndrome, culminating in an international consensus statement of ‘post-operative paediatric cerebellar mutism syndrome’ (pCMS) as the preferred terminology [14]. In earlier reports, incidence of pCMS was thought to be as low as 8% [28, 29], although more recently, robust prospective studies [12] have shown that around one quarter of children undergoing infratentorial craniotomy for tumour resection will develop some form of pCMS. Twelve of 56 patients (21.4%) developed pCMS in this series, a figure comparable with that of other reports [11, 12].

**Fig. 2:**
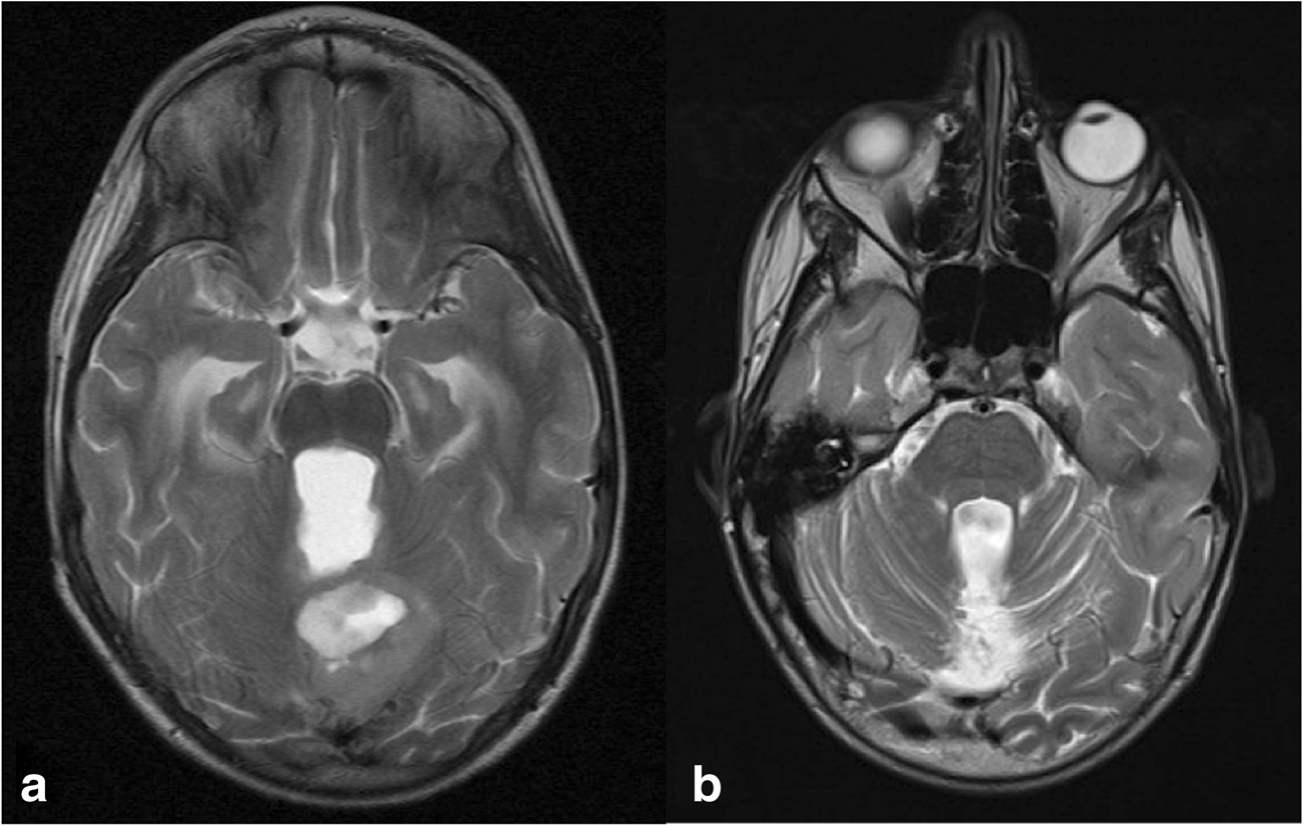
Images from a child who did not develop pCMS. **a** Pre-operative axial T2-weighted MRI showing cystic midline cerebellar tumour causing distortion of the SCPs. **b** Post-operative axial T2-weighted MRI showing normal architecture and signal of both SCPs

**Fig. 3:**
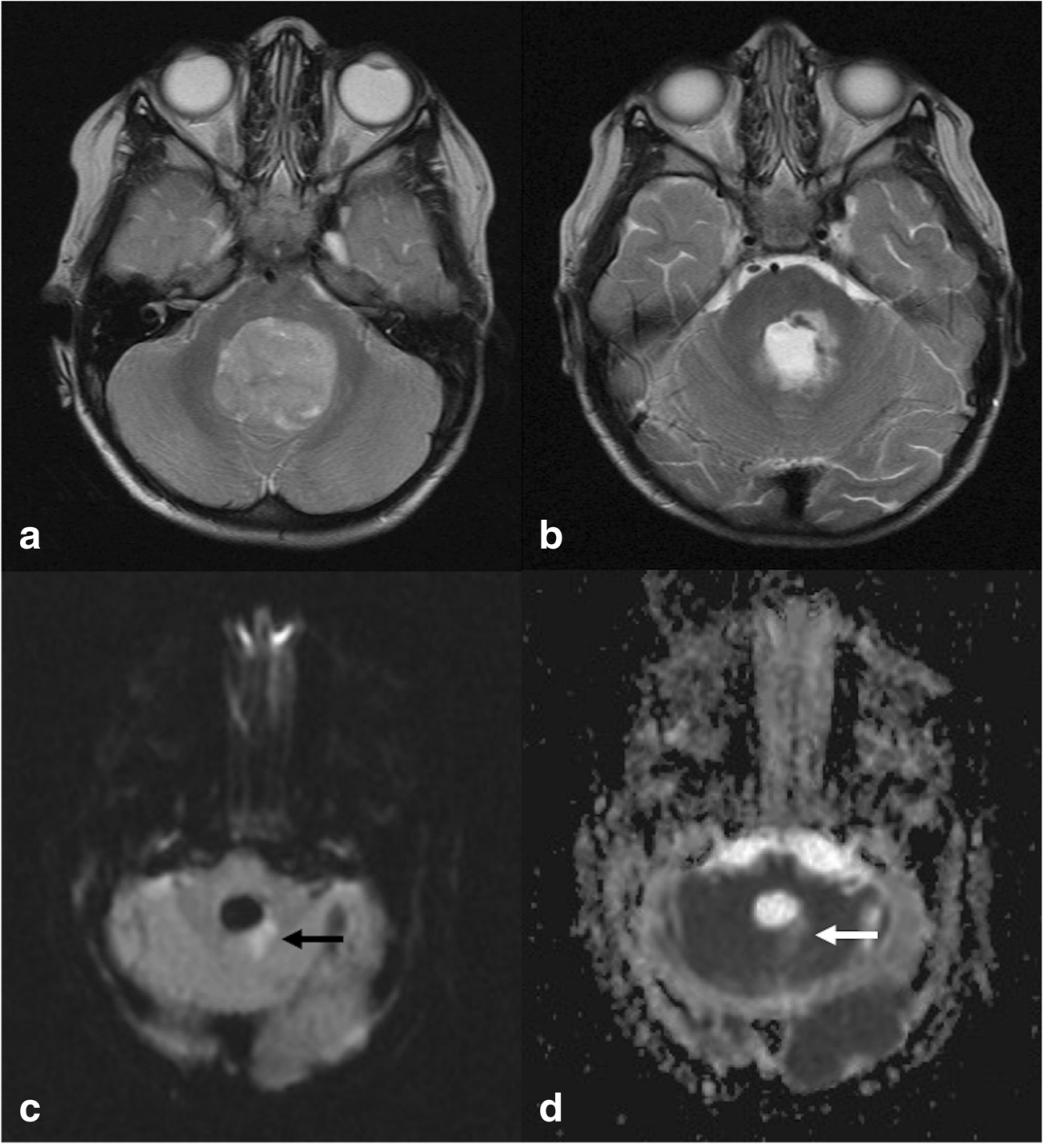
Images from a child who developed pCMS. **a** Pre-operative axial T2-weighted MRI showing tumour occupying the fourth ventricle, with distortion and signal change in the left dentate nucleus region. **b** Early post-operative axial T2-weighted MRI showing left-sided dentate nucleus signal change (which also extended to the left SCP on other slices). **c** Post-operative DWI (b = 1000) showing left-sided dentate nucleus signal change (black arrow), also evident in **d** ADC map at same position (white arrow)

**Fig. 4:**
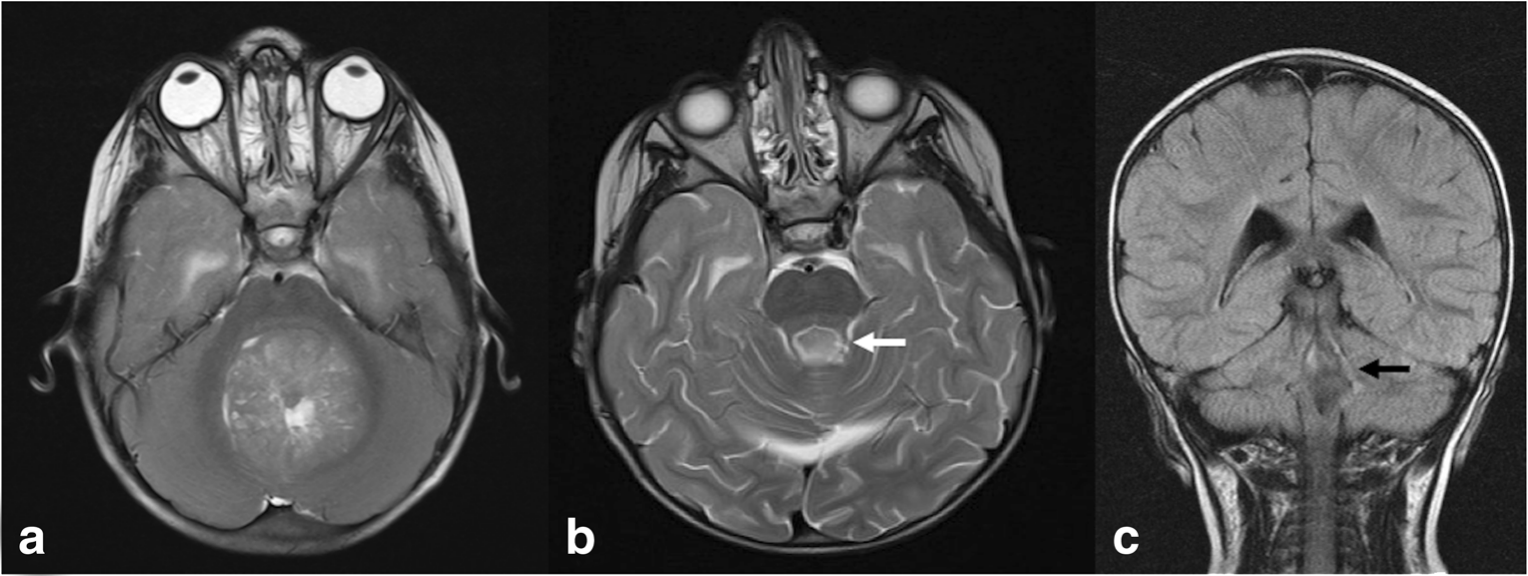
Images from a child who developed pCMS. **a** Pre-operative axial T2-weighted MRI showing tumour occupying the fourth ventricle, with distortion of the left SCP and dentate nucleus region. **b** Early post-operative axial T2-weighted MRI showing left SCP disruption and signal change (white arrow). **c** Delayed post-operative coronal FLAIR MRI showing persistent left-sided SCP signal change (black arrow)

In line with the findings of other groups, this series shows no significant association between pCMS and gender [12] or size of tumour [23], although there was some evidence that younger age at diagnosis carries an increased incidence of pCMS. Figures 2, 3 and 4 show representative scans from the present cohort. With regard to determining rostrocaudal tumour location in the fourth ventricle, attempts to replicate the ratio of tumour above/below the midpoint of the fourth ventricle, used previously [17], was not possible as many tumours obliterated the fourth ventricular cavity, making estimation of its midpoint impossible. Consistent with the initial reports of pCMS, several studies have shown that tumour histology of medulloblastoma has a strong association with its occurrence [19, 24, 30]. Other posterior fossa tumours often arise in, and remain restricted to, other locations within the posterior fossa, such as the cerebellar hemispheres, cerebellar vermis or cerebellopontine angle. In the context of the current understanding of the aetiology of pCMS, the contribution of these structures to its development is less clear. We therefore decided to limit our study to a consistent cohort of medulloblastomas, which typically arise within the fourth ventricle (see Figs. 3 and 4).

Efforts have been made to predict the development of pCMS from pre-operative structural MRI scans by retrospective review [28, 31]. Many early clinical series on posterior fossa syndrome were unable to consistently demonstrate any salient pre-operative radiographic features, beyond brainstem infiltration and compression, which has since been confirmed in several studies [11, 12, 23, 24, 32, 33]. In a recently developed pre-operative risk stratification tool, based on a cohort of children and young adults with unselected posterior fossa tumours recruited from two institutions, three of four variables included in the model were pre-operative MRI features (cerebellar hemisphere tumour location, middle cerebellar peduncle and dentate nucleus involvement) [34]. Tumour location in the cerebellar hemisphere was found to be protective against pCMS. As referred to above, the cohort reported here includes solely medulloblastomas, which are more likely to arise in the midline, thus altering the baseline risk of pCMS developing. The middle cerebellar peduncle carries afferent connections to the cerebellum, and its role in the development of pCMS is at present less well-supported than that of the SCP—for this reason, we chose not to include it in our analysis. Our findings do implicate the dentate nucleus based on delayed post-operative changes, but we were unable to show its utility in predicting pCMS from pre- or early post-operative scans. The final component of the predictive model is an age over 12.4 years, yet our results, and those of other groups [32], indicate that a younger age may be associated with the development of pCMS. Prospective validation of this model is awaited, yet it remains the first of its kind to use strictly pre-operative findings to inform the highly sensitive discussions of consent and complications of posterior fossa tumour surgery. Pre-operative language impairment is another clinical feature which, if present alongside the aforementioned pre-operative structural MRI changes, has been shown to be associated with the development of pCMS [35, 36]. Evaluations of patients’ neurocognitive and linguistic status pre-operatively may further assist in risk prediction on an individualised basis.

Whilst it may be difficult to predict development of pCMS from pre-operative imaging, early post-operative scans often reveal relevant abnormalities in those with pCMS, in particular oedema of the superior and middle cerebellar peduncles [11, 28], the former constituting a major efferent pathway of the cerebellum. Our results in Tables 3 and 4 confirm this strong association between signal change in the SCP and pCMS on post-surgical structural MRI sequences. Intra-operative post-resection DWI sequences have shown that in some patients, diffusion-weighted signal change in pECP structures can be interpreted as a highly specific risk factor for development of pCMS [37]. Of 28 DWI scans reviewed in this series, diffusion abnormalities were identified in 10; 7 of these involved the pECP, and pCMS developed in 6 of these 7 patients. This study benefited from immediate post-resection imaging in an intra-operative MRI suite, allowing very early changes in signal to be detected. Our methodology is limited by the heterogeneous interval between surgery and scanning, which may have led to a dilution in any observed effect, thus we were unable to confirm the findings of Avula et al., although Fig. 3 shows representative post-operative DWI abnormalities.

Damage to the dentate nucleus (see Fig. 3) has long been thought to be crucial for the development of pCMS, since the incidental description of reversible mutism as a complication in patients undergoing stereotactic surgery [38]. In our study, pre-operative or early post-operative T2-weighted signal change of the dentate nucleus was not associated with pCMS. This result is consistent with the findings of Morris et al., who showed that uni- or bilateral dentate nucleus signal change at any time point is not mandatory for development of pCMS. In contrast to this, we found significant differences between groups in dentate nucleus signal on delayed scans. This might be due to a change in the underlying neural circuitry as has been hypothesised [21], although this fails to explain the absence of red nucleus involvement on post-operative scans.

The phenomenon of hypertrophic olivary degeneration (HOD) has recently been shown to be strongly associated with pCMS [39, 40]. This is a radiological sign seen on delayed post-operative imaging of increased signal (most conspicuous on proton density weighted sequences) in the inferior olivary nucleus, the terminal station of the so-called triangle of Guillain-Mollaret (see Fig. 1). It is thought to result from trans-synaptic degeneration along the dentato-rubro-thalamo-cortical tract, which contributes to the afferent limb of the aforementioned circuit. Its utility as a clinical indicator, however, is limited by virtue of its being an inherently post-operative phenomenon. As such, it may be employed as an a posteriori validation tool of pCMS, but never as a predictor. There may be several reasons why our results did not demonstrate olivary signal change on delayed imaging in this cohort of patients. Firstly, proton density images are not acquired on a routine clinical basis in our institution, thus limiting the sensitivity of detecting such changes. Secondly, the extended follow-up time point of many patients (median 31 months) may have meant that the olivary changes had reached their nadir, i.e. olivary *atrophy*, again impairing detection of any relevant change. Thirdly, not all patients will have been imaged at the same point within the development of olivary changes on imaging, thus mitigating any potential group effects.

In the search for structural and functional aberrancies in the brains of children with pCMS, increasingly advanced MRI sequences, over and above the conventional techniques described in this paper, are being utilised. These include tractography to reconstruct relevant white matter pathways, generated from diffusion MRI data using the tensor model, a technique first described over 20 years ago [41]. In a landmark paper, Morris et al. [17] were the first to deploy this technique in patients with pCMS, with post-operative diffusion tensor imaging showing reduced fractional anisotropy (a measure of the coherence of diffusion directionality) in the pECP as well as widespread supratentorial regions. Since then, a number of studies have shown disruption of cerebello-cerebral circuitry in cerebellar mutism [18–20]. Yet diffusion MRI signal modelling, and its translation into clinical practice, has progressed significantly since 2009 [42], and now utilises state-of-the-art techniques beyond the diffusion tensor, which are believed to more faithfully represent the underlying white matter pathways. It is possible that such approaches may yield further insights into the pathophysiology of pCMS, and may help predict its development, or even contribute to surgical guidance to avoid it.

The authors recognise the limitations inherent in this retrospective work. Firstly, as alluded to above, the nomenclature of pCMS is inconsistent at best [43], although the Posterior Fossa Society’s recent consensus statement will hopefully eliminate such discrepancies in future work [14]. There is only one published clinical scoring system [12] for this variegated syndrome, although it is yet to be validated prospectively. Our approach in this study therefore was pragmatic and based around the core symptom of mutism. It is evident that classification of pCMS remains challenging, especially if performed on a retrospective basis as in this study. We therefore recognise the possibility of selection bias in categorisation of pCMS cases. From a radiological perspective, many of the imaging surrogates implicated in pCMS are qualitatively assessed on conventional MRI, introducing subjectivity into the analysis, but this was mitigated against by having scans reviewed concurrently by two senior neuroradiologists. In addition, there is always some difficulty in recapitulating findings of other studies utilising MRI, as regions of interest are small, and the many parameters of conventional and more advanced acquisition sequences are not standardised between units.

## Conclusions

In a retrospective cohort of 56 children who underwent surgery for medulloblastoma, 12 (21.4%) developed pCMS. The development of pCMS could not be linked to any observable changes on pre-operative conventional MRI scans. Post-operative MRI scans confirm the involvement of the SCP and dentate nucleus in pCMS. Future studies incorporating state-of-the-art advanced MRI sequences are called for in order to elucidate the nature of global connectivity changes in pCMS and their relationship to the increasingly well-defined semiology of this debilitating post-operative condition.
